# A p53/TIAF1/WWOX triad exerts cancer suppression but may cause brain protein aggregation due to p53/WWOX functional antagonism

**DOI:** 10.1186/s12964-019-0382-y

**Published:** 2019-07-17

**Authors:** Pei-Yi Chou, Sing-Ru Lin, Ming-Hui Lee, Lori Schultz, Chun-I Sze, Nan-Shan Chang

**Affiliations:** 1 Institute of Molecular Medicine, National Cheng Kung University, College of Medicine, Tainan, Taiwan 70101 Republic of China; 2 Laboratory of Molecular Immunology, Guthrie Research Institute, Sayre, PA 18840 USA; 3 Department of Cell Biology and Anatomy, National Cheng Kung University, College of Medicine, Tainan, Taiwan 70101 Republic of China; 4 Department of Neurochemistry, New York State Institute for Basic Research in Developmental Disabilities, Staten Island, NY 10314 USA; 5 Graduate Institute of Biomedical Sciences, College of Medicine, China Medical University, Taichung, 40402 Taiwan, Republic of China

**Keywords:** Tumor suppressor, WWOX, p53, TIAF1, Cell migration, Promoter activation

## Abstract

**Background:**

Tumor suppressor WWOX physically binds p53 and TIAF1 and together induces apoptosis and tumor suppression. To understand the molecular action, here we investigated the formation of WWOX/TIAF1/p53 triad and its regulation of cancer cell migration, anchorage-independent growth, SMAD promoter activation, apoptosis, and potential role in neurodegeneration.

**Methods:**

Time-lapse microscopy was used to measure the extent of cell migration. Protein/protein interactions were determined by co-immunoprecipitation, FRET microscopy, and yeast two-hybrid analysis. The WWOX/TIAF1/p53 triad-mediated cancer suppression was determined by measuring the extent of cell migration, anchorage-independent growth, SMAD promoter activation, and apoptosis. p53-deficient lung cancer cell growth in nude mice was carried out to assess the tumor suppressor function of ectopic p53 and/or WWOX.

**Results:**

*Wwox*-deficient MEF cells exhibited constitutive Smad3 and p38 activation and migrated individually and much faster than wild type cells. TGF-β increased the migration of wild type MEF cells, but significantly suppressed *Wwox* knockout cell migration. While each of the triad proteins is responsive to TGF-β stimulation, ectopically expressed triad proteins suppressed cancer cell migration, anchorage-independent growth, and SMAD promoter activation, as well as caused apoptosis. The effects are due in part to TIAF1 polymerization and its retention of p53 and WWOX in the cytoplasm. p53 and TIAF1 were effective in suppressing anchorage-independent growth, and WWOX ineffective. p53 and TIAF1 blocked WWOX or Smad4-regulated SMAD promoter activation. WWOX suppressed lung cancer NCI-H1299 growth and inhibited splenomegaly by inflammatory immune response, and p53 blocked the event in nude mice. The p53/WWOX-cancer mice exhibited BACE upregulation, APP degradation, tau tangle formation, and amyloid β generation in the brain and lung.

**Conclusion:**

The WWOX/TIAF1/p53 triad is potent in cancer suppression by blocking cancer cell migration, anchorage-independent growth and SMAD promoter activation, and causing apoptosis. Yet, p53 may functionally antagonize with WWOX. p53 blocks WWOX inhibition of inflammatory immune response induced by cancer, and this leads to protein aggregation in the brain as seen in the Alzheimer’s disease and other neurodegeneration.

**Electronic supplementary material:**

The online version of this article (10.1186/s12964-019-0382-y) contains supplementary material, which is available to authorized users.

## Background

WW domain-containing oxidoreductase, designated WWOX, FOR or WOX1, is a 46-kDa tumor suppressor protein ([1–6]; reviews). *WWOX* gene is located on a common fragile site FRA16D on chromosome ch16q23.3–24.1, encompassing a million bases [1–8]. Loss of WWOX protein occurs as a result of genetic alterations [1–6], promoter hypermethylation [9–12], and translational blockade [13], and this may be associated with cancer development [1, 2, 4–6]. Significant downregulation of WWOX protein is frequently seen in metastatic cancer cells [1–6]. Loss of WWOX upregulates the JAK2/STAT3 pathway that drives cancer metastasis in triple negative breast cancer cells [14]. Also, loss of WWOX in ovarian cancer cells acquires enhanced migration and metastasis due to altered interactions between integrin α3 and fibronectin [15]. WWOX suppresses the expression of RUNX2 and thereby blocks the invasion and metastasis of osteosarcoma and lung cancer cells [16, 17].

Despite its connection with cancer, WWOX indeed plays a critical role in neural development and neurodegeneration. *WWOX* gene has recently been determined as a risk factor for Alzheimer’s disease [3, 18]. Null mutations of *WWOX*/*Wwox* gene cause severe neural diseases (e.g. epilepsy, microcephaly, retinal degeneration, and ataxia), metabolic disorders (including lipid, cholesterol and glucose metabolism), and early death in the newborns [1, 3, 19, 20]. No spontaneous tumor growth is shown in the newborns of humans and rats, suggesting that WWOX primarily maintains the physiology of normal tissues and organs. WWOX participates in the neural development [3, 21]. WWOX deficiency leads to rapid protein aggregation to cause neuronal damage and death in vivo [3, 22–26]. For example, shortly after birth for 15 days, *Wwox* knockout mice develop brain protein aggregation, including TRAPPC6AΔ (Trafficking protein particle complex 6A delta) [24–27], TIAF1 (TGFβ1-Induced Anti-Apoptotic Factor 1) [23, 24, 28], SH3GLB2 (SH3 Domain Containing GRB2 Like, Endophilin B2) [26], tau [3, 26] and amyloid β [3, 26], become aggregated in the brains of newborn [22–28]. Loss of WWOX probably induces conformational changes of the aforementioned proteins leading to aggregation.

Transiently overexpressed WWOX with Tyr33 phosphorylation (pY33-WWOX) induces apoptosis [1, 3, 6, 29–37]. pY33-WWOX also maintains the normal physiology of cells [1, 3, 6]. pY33-WWOX works together with p53, Hyal-2 and Smad4 to induce apoptosis [21–23, 34, 35]. In response to UV and cold shock, WWOX, p53 and NOS2 (nitric oxide synthase 2) generate a novel type of cell death, termed bubbling cell death, in many types of cells [35–37]. When overexpressed, ectopic Hyal-2/WWOX/Smad4 signaling complex causes bubbling cell death in response to high-molecular-weight hyaluronan of 2–4 million Daltons [35, 37]. In contrast, hyaluronan increases the binding and signaling of p53/WWOX/Smad4 for leading to membrane blebbing, but without causing cell death [35, 37].

During the early stage of cancer development, WWOX is upregulated in the hyperplasia tissues [13, 38]. Activated pY33-WWOX is rapidly upregulated in 24 h during the acute phase of UVB irradiation-induced skin squamous cell carcinoma (SCC) in hairless mice [13]. pY33-WWOX probably exerts its tumor suppressor function to limit cancer progression. pY33-WWOX disappears in 3 months in the UVB-treated mice as SCC continuously develops, even though the mRNA coding for *Wwox* is still present. Similarly, pY33-WWOX upregulation is observed during breast cancer progression to a premetastatic state [38]. Estrogen induces the upregulation and activation of WWOX [38]. Perhaps, upregulated pY33-WWOX can be regarded as a marker for the very early stage of cancer progression.

We have reported the protein complex of WWOX, p53 and TIAF1 triad is a potential axis of tumor suppression [23, 28]. Here, we examined whether the protein triad suppresses anchorage-independent growth, blocks cell migration, inhibits SMAD promoter activation, and causes apoptosis. We determined the role of p53 phosphorylation at Ser46 in contributing to the apoptotic function of the triad. The kinetics of the triad formation was examined by FRET (Förster resonance energy transfer) microscopy, co-immunoprecipitation, and yeast two-hybrid analysis [22–26, 28–32, 34–39]. Without activation, p53 cannot bind TIAF1 [40]. Intriguingly, activated p53 binds TIAF1 and then together with WWOX to form a stabilized triad. Among p53 isoforms, Δ133p53γ is most potent in suppressing the migration of WWOX-negative MDA-MB-231 cells, which correlates with its activation of SMAD promoter. Notably, p53 may functionally antagonize with WWOX. p53 blocks WWOX inhibition of inflammatory immune response induced by cancer, and this leads to protein aggregation in the brain as seen in the Alzheimer’s disease (AD) and other neurodegeneration.

## Methods

### Cell lines and cell culture

Cell lines used in this study included human breast MCF-7 and MDA-MB-231 cancer cells [38], human prostate DU145 cells [35], human monocytic U937 cells [35], human lung p53-deficient NCI-H1299 [35], murine L929 fibroblasts [29–31], monkey kidney SV40 virus-transformed COS7 fibroblasts [31, 35], and primary mink lung epithelial Mv1Lu cells [40, 41] (American Type Culture Collection). Mouse embryonic fibroblast (MEF) for *Wwox* wild type, heterozygous and knockout cells were generated and maintained in RPMI-1640 medium supplemented with 10% fetal bovine serum [25]. All the cells were cultured at 37 °C in an incubator with 5% CO_2_ / atmosphere.

### Chemicals, antibodies, Western blotting, immunofluorescence microscopy, and co-immunoprecipitation

Recombinant TNFα, TGF-β1 and -β2 proteins were from PeproTech. Phalloidin for F-actin staining was from Invitrogen. Antibodies against ERK (extracellular signal–regulated kinase), pERK (phosphorylated ERK), BACE (β-secretase), WWOX, p53, BECN-1 (Beclin-1), IκBα (inhibitor of nuclear factor kappa B alpha), *Foxp3* (Forkhead box P3), AIF (apoptosis inducing factor) and GFP (green fluorescent protein) were from Santa Cruz Biotechnology. Additional commercial antibodies used were against: Aβ (AbD Serotec), APP (EMD Millipore), NFT (neurofibrillary tangles; Invitrogen), and α-tubulin (Sigma-Aldrich) [21–25]. Homemade antibodies against WWOX were also used [13, 21–25]. Antibodies were used for Western blotting analysis and immunofluorescent microscopy, as described [21–25, 29–31]. Where indicated, binding of WWOX with TIAF1 in response to TNFα and TGF-β1 were determined by co-immunoprecipitation using specific antibodies, as described [29–31, 41].

### cDNA constructs and electroporation

We have first isolated the murine full-length *Wwox* cDNA [29]. Full-length dominant-negative *Wwox* construct was mutated on Lys28 to Thr28 and Asp29 to Val29 in the first WW domain (dn*Wwox*) [30]. Another dominant negative construct contained the *N*-terminal WW domains with the same mutation (dn*ww*) [30]. A wild type TIAF1 [40–42] and 3 TIAF1 dominant negative constructs were made: TIAF1 (S6G) and TIAF (S37G) with Ser6 mutated to glycine and Ser37 to glycine, respectively, and TIAF1 (S68A) with Ser68 mutated to alanine. Ser37 is a confirmed phosphorylated site [22–24]. These three mutation sites were the predicted phosphorylation sites in TIAF1 protein by using NetPhos 2.0 Server (Technical University of Denmark). Other constructs used were p53 and p53(S46G) [23, 29–31], and eight p53 isoforms [43–46]. p53 isoforms were kind gifts of Dr. JC Bourdon of the University of Dundee. All of the constructs used were made in pEGFP-C1, pECFP-C1 and pDsRed vectors (Clontech), respectively. *Wwox* siRNA (*Wwox*si) was designed and cloned into pSuppressorNeo vector (Imgenex) [47]. Designed primers for WWOXsi#1 and #2 siRNAs, targeting a common DNA sequence in human/murine WWOX and another DNA sequence in human WWOX, respectively, were made [47]. Cells were electroporated twice with the indicated DNA constructs (200 V, 50 ms) and cultured in medium containing 10% FBS overnight prior to carrying out experiments. While indicated, liposome-based GeneFECTOR (Venn Nova) was used to transfect cells with the expression constructs.

### Cell migration assay, promoter activation, and time-lapse microscopy

Cell migration assay was performed as described [24]. A culture insert (*ibidi*) was placed onto a 35 mm dish, and an equal number of cells (70 μl, 4 × 10^5^ cells) were seeded into the two reservoirs of the same insert, so there would be a 500 ± 50 mm gap. After overnight incubation at 37 °C/5% CO2, the insert was gently removed and the medium was changed to serum-free, or contain 2% FBS to minimize cell proliferation. The cell migration was imaged at an indicated time interval for 24 to 48 h using a NIKON TE2000-U microscope [23–26]. Cell migration was analyzed either by counting the migrating cell numbers or by measuring the migrating cell areas. An inverted Olympus IX81 fluorescence microscope was used for carrying out time-lapse microscopy [34–37]. Cell migration rate was measured by cell migrating distance versus time. Single cell moving path was tracked using the NIH Image J manual tracking and chemotaxis and migration tool.

### Tri-molecular FRET microscopy

The kinetics of tri-molecular protein/protein binding interactions was carried out by FRET microscopy [35, 37, 39]. Experiments were designed to let the FRET energy transfer from WWOX-ECFP to TIAF1-EGFP, and finally to p53-DsRed. COS7 cells were transiently overexpressed with ECFP-WWOX, EGFP-TIAF1, and DsRed-p53. In negative controls, cells were transfected with ECFP, EGFP, and DsRed. Following culturing overnight, cells were treated with Prima-1 (10 μM), an activator of p53 activator, for 0, 30 and 60 min, followed by fixing with 4% paraformaldehyde. Similar experiments were carried out by treating cells with TGF-β1 (10 ng/ml). FRET microscopy was performed using an inverted fluorescence microscope (Nikon Eclipse TE-2000 U), and data analyzed as described [35, 37, 39]. The FRET images were corrected for background fluorescence from an area free of cells and spectral bleed-through. The spectrally corrected FRET concentration (FRETc) was calculated by Youvan’s equation (using a software program Image-Pro 6.1, Media Cybernetics): FRETc = [fret − bk(fret)] − cf.(don) × [don − bk(don)] − cf.(acc) × [acc − bk(acc)], where fret = fret image, bk = background, cf. = correction factor, don = donor image, and acc = acceptor image. The equation normalizes the FRET signals to the expression levels of the fluorescent proteins.

### Cytoplasm-based yeast two-hybrid analysis for protein/protein binding interactions

To investigate whether WWOX binds TIAF1 in vivo, Ras rescue-based yeast two-hybrid analysis (CytoTrap; Stratagene) was performed [29–31, 34, 35]. In brief, binding of a cytosolic Sos-tagged bait protein to a cell membrane-anchored target protein (tagged with a myristoylation signal) leads to activation of the Ras signaling pathway in yeast. This activation allows mutant yeast cdc25H to grow in 37 °C using a selective agarose plate containing galactose. Without binding, yeast cells fail to grow at 37 °C. Target constructs made in a pMyr vector (with the myristoylation signal) were murine TIAF1 and human p53. Bait constructs made in a pSos vector (tagged with an *N*-terminal Sos protein) were murine WWOX, WWOXww (the first WW domain), WWOX(Y33R), and WWOXsdr (the entire SDR domain). Additionally, self-binding of MafB (in both pMyr and pSos) was regarded as a positive control, and empty pSos versus empty pMyr as a negative control.

### Cell cycle analysis

Cells were electroporated with indicated EGFP-tagged plasmids and cultured overnight. The electroporation efficiency was confirmed by fluorescence microscopy. The condition media were then harvested, and the cells collected by trypsin and centrifugation (4000 rpm, 10 min). Cells were washed once with PBS and then were fixed in 70% ethanol overnight. After overnight fixation, cells were then washed once with PBS and stained with propidium iodide (PI) solution (2 μg/ml PI, 10 μg/ml RNase A in PBS) for 30 min at room temperature. Cell cycle analysis was performed by flow cytometry (BD) [23, 24, 29–31].

### Cell proliferation assay

5 × 10^4^ cells per well were seeded in 12-well microtiter plates, cultured overnight in medium containing 10% FBS, and then trypsinized. The cell number was counted using hemocytometer at time point 0, 6, 18, 24, and 48 h.

### Soft agarose colony survival assay

Adherence-independent cell growth or transforming growth was performed in a soft agarose colony survival assay [40]. MDA-MB-231 or indicated cells were electroporated with expression constructs of p53-DsRed, WWOX-ECFP, and/or EGFP-TIAF1. These cells were plated at a density of 3 × 10^4^ cells/35-mm dish in triplicate in RPMI 1640, 10% fetal bovine serum, 0.8% agarose, and 10 mM HEPES. Dishes were incubated in a humidified CO2 incubator at 37 °C for 3 weeks. Live colonies were stained with the MTS proliferation reagent (Promega) and counted. In controls, cells were treated with DMEM medium or subjected to electroporation with medium only.

### Cancer cell growth in nude mice

p53-deficient NCI-H1299 lung cancer cells were transiently overexpressed with p53-DsRed and/or WWOX-CFP, or EGFP only using liposome-based GeneFECTOR (Venn Nova). Nude mice received subcutaneous injections of these cells twice on both sides of the flanks, followed by measuring the tumor sizes daily, as described [26].

### Statistical analysis

Data were analyzed by Student’s *t* test among controls and tested groups using Microsoft excel. Data were expressed as mean ± standard deviation, where *p* < 0.05 was considered significant.

## Results

### Altered signaling in *Wwox* knockout MEF cells

**Fig. 1:**
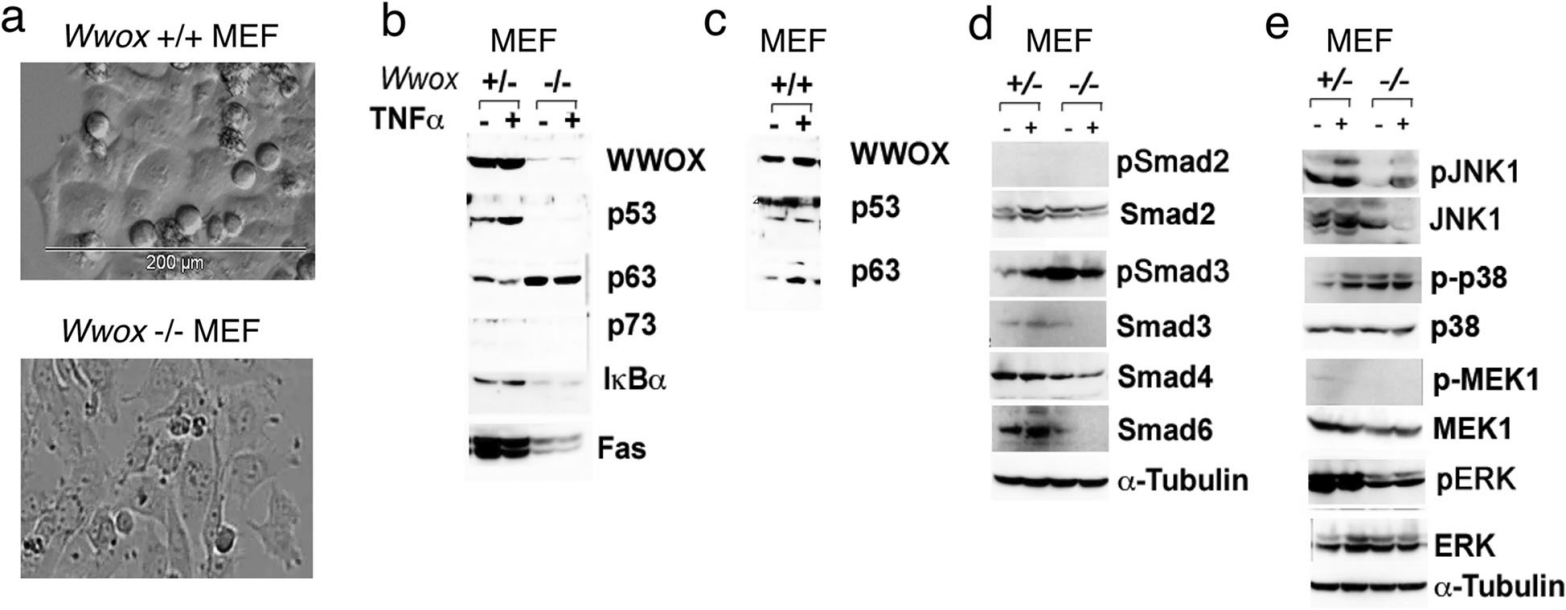
Altered signaling in *Wwox* knockout MEF cells. **a** Wild type MEF *Wwox*^+/+^ cells tightly merge with each other and appear squamous. Dividing cells at metaphase are roundish. Similar observations were shown for heterozygous *Wwox*^+/−^ MEF cells. Knockout *Wwox*^−/−^ MEF cells exhibit loose cell-cell contacts. **b**, **c** *Wwox* MEF cells were treated with TNFα for 1 h. Protein expression in the TNF pathway is shown. **d** Under similar conditions, protein expression in the SMAD pathway is shown. Smad3 is constitutively phosphorylated or activated in the knockout cells. **e** Expression of proteins in the MAPK pathway is shown. P38 is constitutively activated

*Wwox* gene knockout mice and wild type *Wwox*^+/+^, knockout *Wwox*^−/−^, and heterozygous *Wwox*^+/−^ mouse embryonic fibroblasts (MEF) were established [25]. Wild type MEF cells tightly merge with each other and appear squamous (and roundish for dividing cells) (Fig. 1a). *Wwox* knockout MEF cells appear similarly to that of the wild type cells, and have loose intercellular connections (Fig. 1a). Compared to the heterozygous *Wwox* MEF cells, knockout cells have a significant reduction in expression of endogenous p53, IκBα and Fas (Fig. 1b). TNFα did not restore the protein levels during treatment of knockout cells for 1 h (Fig. 1b). Interestingly, knockout cells have a higher expression of p63 than the heterozygous cells (Fig. 1b). The protein levels for p53, p63 and WWOX in the wild type cells are shown (Fig. 1c). Smad3 of the SMAD pathway was constitutively phosphorylated (or activated) in the knockout cells (Fig. 1d). Also, in the knockout cells, inhibitor Smad6 was significantly downregulated (Fig. 1d). Smad2, 3 and 6 were responsive to TNFα-mediated upregulation in the heterozygous cells, but not in the knockout cells (Fig. 1d). In the MAPK pathway, p38 is constitutively activated in the knockout cells (Fig. 1e). TNFα induced the activation of JNK1 in both heterozygous and knockout cells (Fig. 1e). Together, compared to the heterozygous cells, knockout MEF cells exhibit many aberrant signaling pathways.

### WWOX is a potent inhibitor of cell migration

**Fig. 2:**
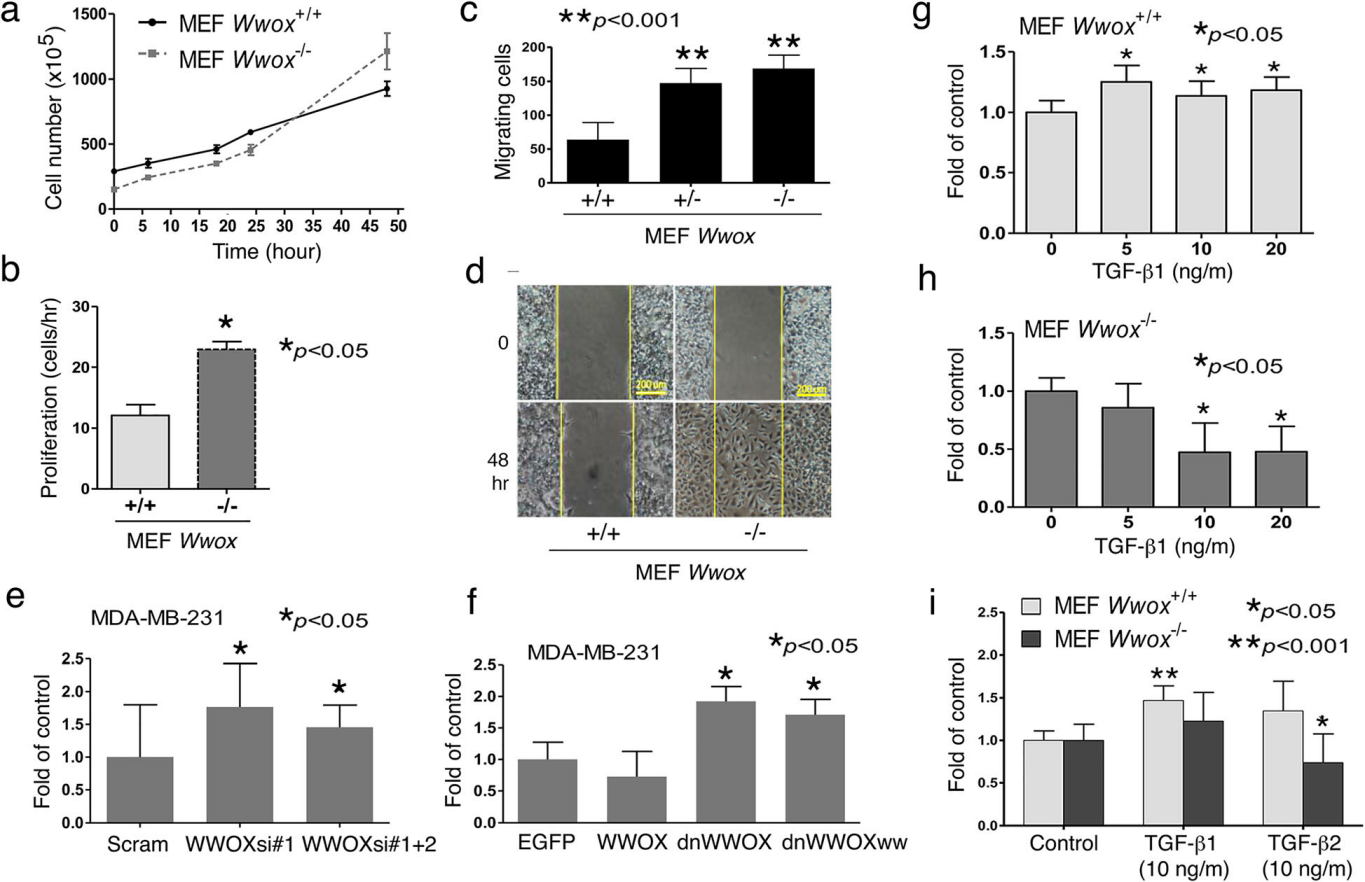
WWOX inhibits cell proliferation and migration. **a** MEF cells were seeded on a 24-well plate and cultured for indicated periods. Live cell numbers were counted using a hemocytometer. **b** *Wwox* knockout MEF cells had a higher proliferation rate than wild type cells during culturing for 48 h (Mean ± S.D., *n* = 3, Student’s *t* test, *p* < 0.05). **c** *Wwox*^−/−^ and *Wwox*^+/−^ MEF cells migrated significantly faster than wild type *Wwox*^+/+^ cells (*n* = 3, Student’s *t* test). **d** Wild type cells migrated collectively, while WWOX-deficient cells migrated individually (see Additional file 2: Video S1 and Additional file 3: Video S2). **e**, **f** Breast MDA-MB-231 cancer cells were transfected with the indicated constructs by electroporation. Transiently overexpressed WWOX suppressed cell migration, whereas dnWWOX and WWOX siRNA constructs enhanced the migration. Dn: dominant negative; WWOXww: WW domain of WWOX; Scram: scramble control; WWOXsi#1 and #2: siRNAs targeting human/murine WWOX (*n* = 3, Student’s *t* test) [47]. **g**, **h** During treatment for 48 h, TGF-β1 promoted wild type cell migration, but suppressed *Wwox* knockout cell migration in a dose-dependent manner (*n* = 3, Student’s *t* test). **i**. Both TGF-β1 and TGF-β2 promoted wild type MEF cell migration during treatment for 48 h. TGF-β2 is more effective in suppressing *Wwox* knockout cell migration than TGF-β1 (*n* = 3, Student’s *t* test)

Cell proliferation assay was performed to examine the doubling time of wild type and *Wwox* knockout MEF cells. Under normal culture condition (medium containing 10% FBS), the proliferation rate of wild type MEF cells was significantly slower than the knockout MEF cells (Fig. 2a and b). Both *Wwox* knockout and heterozygous MEF cells migrated significantly faster than the wild type cells (Fig. 2c and d). Wild type cells migrated collectively, while WWOX-deficient cells migrated individually (Fig. 2d and Additional file 1: Figure S1 for the enlarged image; Additional file 2: Video S1 and Additional file 3: Video S2).

Metastatic breast cancer MDA-MB-231 cells were used in the cell migration assay. MDA-MB-231 is a triple negative breast cancer cell line, which lacks the expression of estrogen receptor (ER), progesterone receptor (PR) and HER-2, and has a very low level of WWOX due to promoter hypermethylation. MDA-MB-231 cells migrate individually. In contrast, ER^+^ and WWOX^+^ breast MCF-7 cells migrate collectively. Transiently overexpressed WWOX suppressed the migration of MDA-MB-231 cells, whereas inhibition of WWOX by dominant negatives (K28 T/D29V mutation in the full length WWOX or WW domains only) [30, 31], or by small interfering RNA (WWOXsi) [47], significantly enhanced the cell migration (Fig. 2d and e). Similarly, we examined the migration of WWOX-negative MDA-MB-435 s and WWOX-positive L929 (or L929 s) fibroblasts and breast MCF7 cancer cells. Without WWOX, cells migrated faster than those of WWOX positive cells (Additional file 1: Figure S2). WWOX appears to be functionally deficient in MCF7 cells, as these cells effectively migrated similarly to the WWOX-negative MDA-MB-435 s and MDA-MB-231 cells (Additional file 1: Figure S2). L929 s cells are sensitive to tumor necrosis factor (TNF)-mediated apoptosis [29].

### TGF-β1 enhances migration of *Wwox*^+/+^ MEF cells but suppresses that of *Wwox*^−/−^ MEF cells

Transforming growth factor-β1 (TGF-β1) has both tumor suppressive and tumor promoting functions [48]. In the early stage of tumor progression, TGF-β acts as a tumor suppressor by inducing apoptosis and inhibiting tumor cell growth, due in part to its activation of the proapoptotic WWOX and Smad4 [33, 34]. In the late stage, metastatic tumor cells have altered TGF-β receptors or Smads in the TGF-β pathway, thereby resulting in TGF-β-mediated cancer growth. TGF-β induces WWOX activation via Tyr33 phosphorylation and nuclear translocation to suppress cancer growth, and that loss of WWOX is found in a majority of metastatic cancer cells [6, 34].

Here, we investigated whether TGF-β affects the cell migration of wild type and *Wwox* knockout MEF cells. TGF-β1 marginally promoted the wild type MEF cell migration (Fig. 2g). However, TGF-β1 suppressed *Wwox* knockout MEF cells migration (Fig. 2h). At 10 ng/ml, TGF-β1 significantly enhanced the migration of wild type cells, but not *Wwox* knockout cells (Fig. 2g, h). TGF-β2 was more effective in suppressing the migration of *Wwox* knockout cells than TGF-β1 (Fig. 2i and Additional file 1: Figure S3). TFG-β1 had no significant effect on the cell proliferation in both wild type and the *Wwox* knockout MEF cells (Additional file 1: Figure S4).

### The TIAF1/WWOX/p53 axis inhibits cell migration

We have reported the presence of the TGF-β-responsive TIAF1/WWOX/p53 complex as a molecular triad in tumor suppression [23, 40–42]. Suppression of TIAF1 by siRNA enhances cancer cell growth and abolishes WWOX-mediated apoptosis [23]. Similarly, p53-mediated apoptosis is blocked by siRNA targeting TIAF1 or WWOX [23]. p53 plays an inhibitory role in cell motility. p53 blocks epithelial-mesenchymal transition (EMT) and cell migration to prevent metastasis [48, 49]. Loss of p53 expression in MEF cells leads to amoeboid-like movement and increased invasive ability [50, 51]. WWOX physically binds p53 [23, 29–31], whereas p53 does not bind TIAF1 [42], suggesting that WWOX is a bridge for the formation of the TIAF1/WWOX/p53 triad to exert tumor suppression. TIAF1 binds and retains Smad2/3/4 in the cytoplasm and blocks Smad-mediated transcriptional activation [23].

**Fig. 3:**
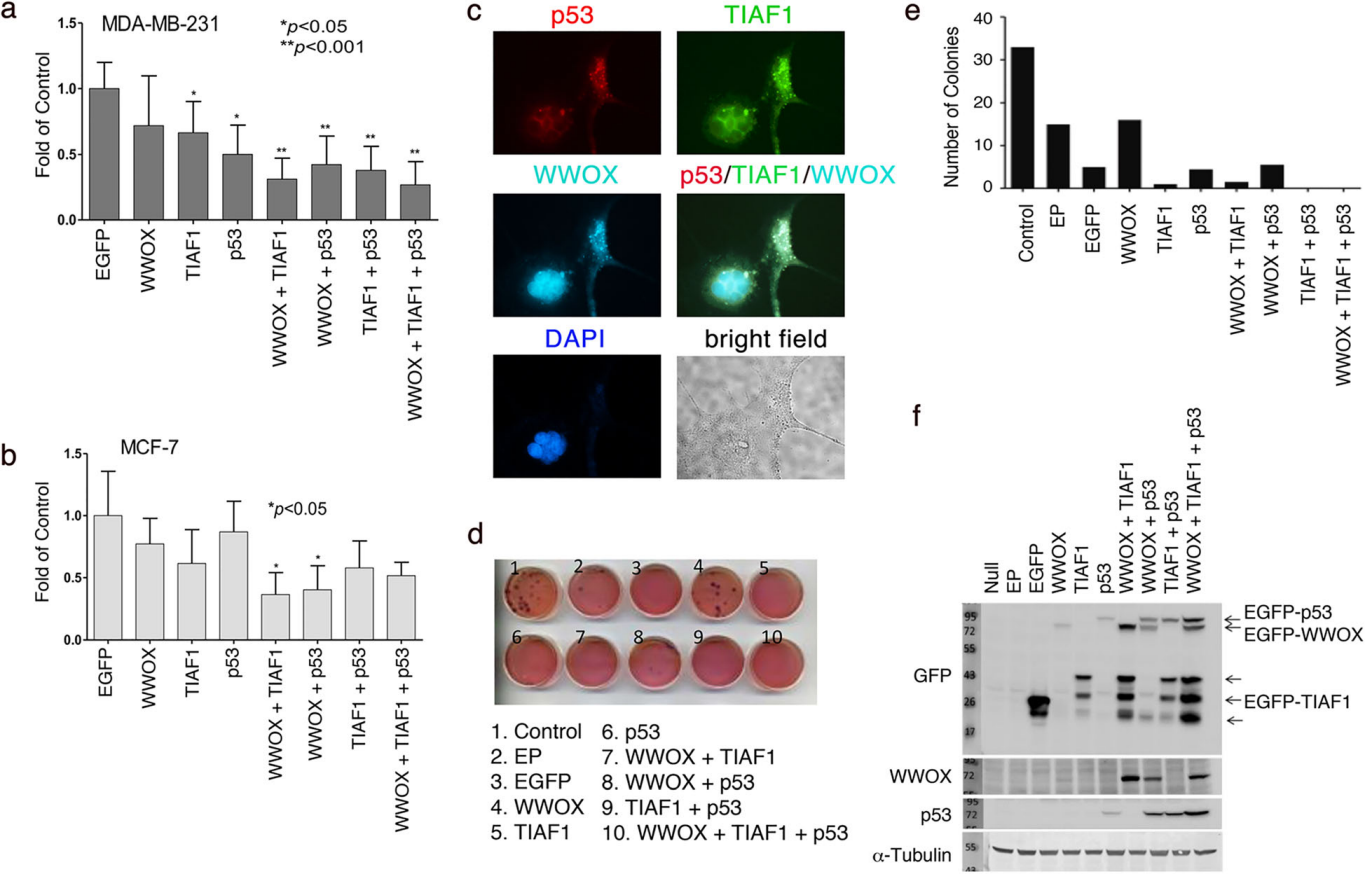
Ectopic WWOX, TIAF1, and p53 suppress cell migration and anchorage-independent cell growth. **a**, **b** Cells were introduced with indicated mammalian plasmids by electroporation. Transient overexpression of WWOX, TIAF1, and/or p53 inhibited the migration of MDA-MB-231 and MCF-7 cell (Mean ± S.D., *n* = 3, student’s *t* test). **c** Colocalization of transiently overexpressed TIAF1 with p53 and WWOX proteins in MDA-MB-231 cells. **d**, **e** In anchorage-independent cell growth, ectopic WWOX, TIAF1 and p53, in combination, dramatically suppressed colony formation of MDA-MB-231. EP, electroporation only. **f** Western blot analysis demonstrated the expressed protein profile in COS7 cells

To determine whether the protein triad controls cell migration, human breast MDA-MB-231 and MCF-7 cells were transiently overexpressed with the cDNA expression constructs for WWOX, TIAF1, and/or p53. Protein expression was examined by a fluorescent microscope. More than 60% of cells were positive for protein expression. Ectopic WWOX, TIAF1, and p53 significantly suppressed the migration of WWOX-deficient MDA-MB-231 and WWOX-positive MCF-7 cells (Fig. 3a and b). A similar extent of migration inhibition was observed by using pairs of expression constructs, including WWOX/p53, WWOX/TIAF1, and TIAF1/p53 (Fig. 3a and b). This is in agreement with our previous observation that ectopic TIAF1/WWOX/p53 triad suppresses the migration of L929 cells [23]. TGF-β, TNF-α, apoptotic stress, or protein overexpression causes TIAF1 undergoes self-aggregation and the aggregates retain Smad proteins to prevent nuclear translocation [22, 23, 28]. Transiently overexpressed TIAF1 underwent aggregation and recruited p53 and WWOX in the aggregates in the cytoplasm (see punctate; Fig. 3c and S5 for enlarged image and additional data), suggesting a likely mechanism for migration inhibition.

### p53 is more potent than WWOX in suppressing anchorage-independent cell growth

In addition, WWOX, TIAF1 and p53, together or in various combinations, inhibited anchorage-independent growth (or transforming growth) of MDA-MB-231 cells, as determined by soft agarose assay (Fig. 3d and e). Compared to p53 and TIAF1, WWOX was much less effective or ineffective in inhibiting the colony formation. Similarly, by using L929 cells, p53 is more potent than WWOX in blocking anchorage-independent growth of L929 cells (Additional file 1: Figure S6). p53ΔS46 has a reduced apoptotic activity and fails to bind WWOX [31], whereas it was potent in blocking the colony formation (Additional file 1: Figure S6). We have previously reported that TIAF1 and p53 are equally potent in suppressing anchorage-independent growth of L929 cells [40].

To further confirm the expression of ectopic proteins, COS7 cells were introduced with GFP-tagged WWOX, TIAF1, and p53, respectively, or in combinations (Fig. 3f). The GFP antibody probed the GFP-tagged proteins, and showed the ectopic protein expressions in COS7 cells. Compared with WWOX expression alone, co-expression of ectopic EGFP-tagged WWOX, TIAF1 and/or p53 upregulated the endogenous WWOX protein expression. Similarly, endogenous p53 was upregulated by ectopic expression of EGFP-tagged p53, WWOX, and/or TIAF1 (Fig. 3f). The reason for using COS7 cells is that this cell line is easy to transfect and ectopic proteins can be readily overexpressed in the cells [29–31].

### Ectopic Δ133p53γ activates SMAD promoter, which correlates with suppression of cancer cell migration

**Fig. 4:**
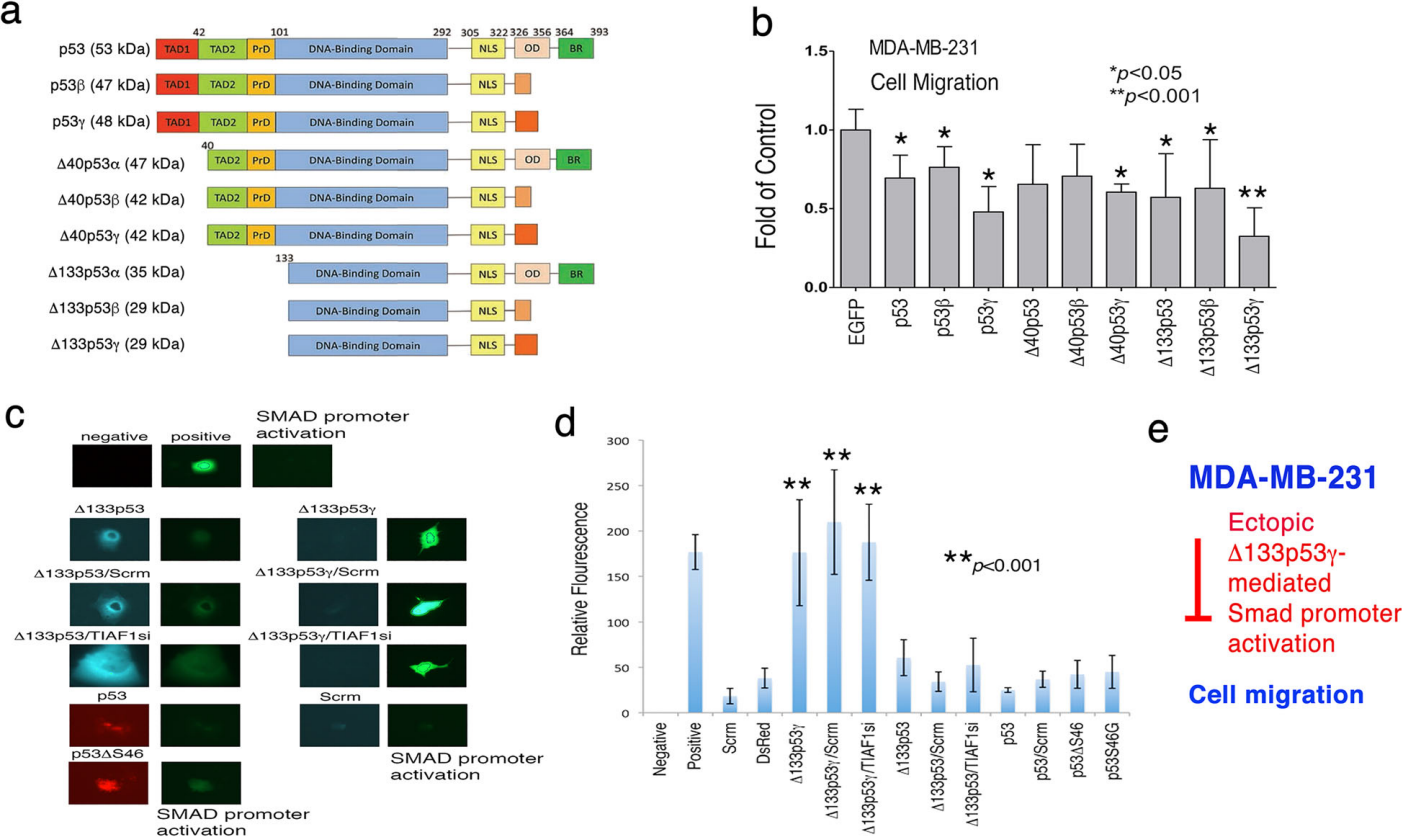
Δ133p53γ is most potent in suppressing cancer cell migration, which correlates with SMAD promoter activation. **a** Nine representative p53 isoforms are shown. There are at least 12 isoforms [43–46]. The schematic graphs for Δ160p53, Δ160p53β and Δ160p53γ are not shown. Full-length p53 possesses two *N*-terminal transactivation acidic domains, a proline-rich domain, a central DNA-binding region, and a *C*-terminal domain, containing a nuclear localization signal, an oligomerization domain, and a basic region. TAD, transactivation domain; PrD, proline domain; NLS, nuclear localization signal; OD, oligomerization domain; BR, basic region. **b** MDA-MB-231 cells transfected with indicated p53 constructs by electroporation. Many p53 constructs significantly suppressed the cell migration (Mean ± S.D., *n* = 3). Δ133p53γ was most effective. **c**, **d** COS7 cells were transiently overexpressed with indicated p53 and isoform constructs, in the presence of an SMAD promoter construct with GFP as a reporter. Ectopic Δ133p53γ significantly induced the SMAD promoter activation (versus TIAF1si and scramble groups; Mean ± S.D., *n* = 3). TIAF1si and scramble constructs did not block the promoter activation. In controls, cells were subjected to mock electroporation with medium (negative), or a positive control construct (positive). **e** A schematic signaling event shows ectopic Δ133p53γ-mediated SMAD promoter activation may lead to inhibition of cell migration

Next, we investigated whether p53 and isoforms control cell migration. p53 has at least 12 isoforms [43–46]. Abnormal expression of p53 isoforms has been reported in several human cancers, such as head and neck cancer and ovarian tumors [42–45], indicating that p53 isoforms participate in tumor progression. MDA-MB-231 cells were transfected with wild type p53 and isoform cDNA expression constructs by electroporation (Fig. 4a). The results showed that many p53 isoforms decreased MDA-MB-231cell migration, and that Δ133p53γ was most effective (Fig. 4b). We have determined that when cells undergo over-activation of the SMAD promoter, apoptosis occurs [34]. Ectopic Δ133p53γ caused the SMAD promoter activation (Fig. 4c and d). In contrast, wild type 53 and Δ133p53 had no effect. TIAF1 binds Smad4 and blocks the SMAD responsive element from activation [23]. Knockdown of TIAF1 by siRNA did not increase Δ133p53γ-mediated SMAD promoter activation (Fig. 4c and d). Together, the observations suggest that ectopic Δ133p53γ activates the SMAD promoter that leads to the restriction of cell migration (Fig. 4e).

### The WWOX/TIAF1/p53 triad induces apoptosis in cancer cells

**Fig. 5:**
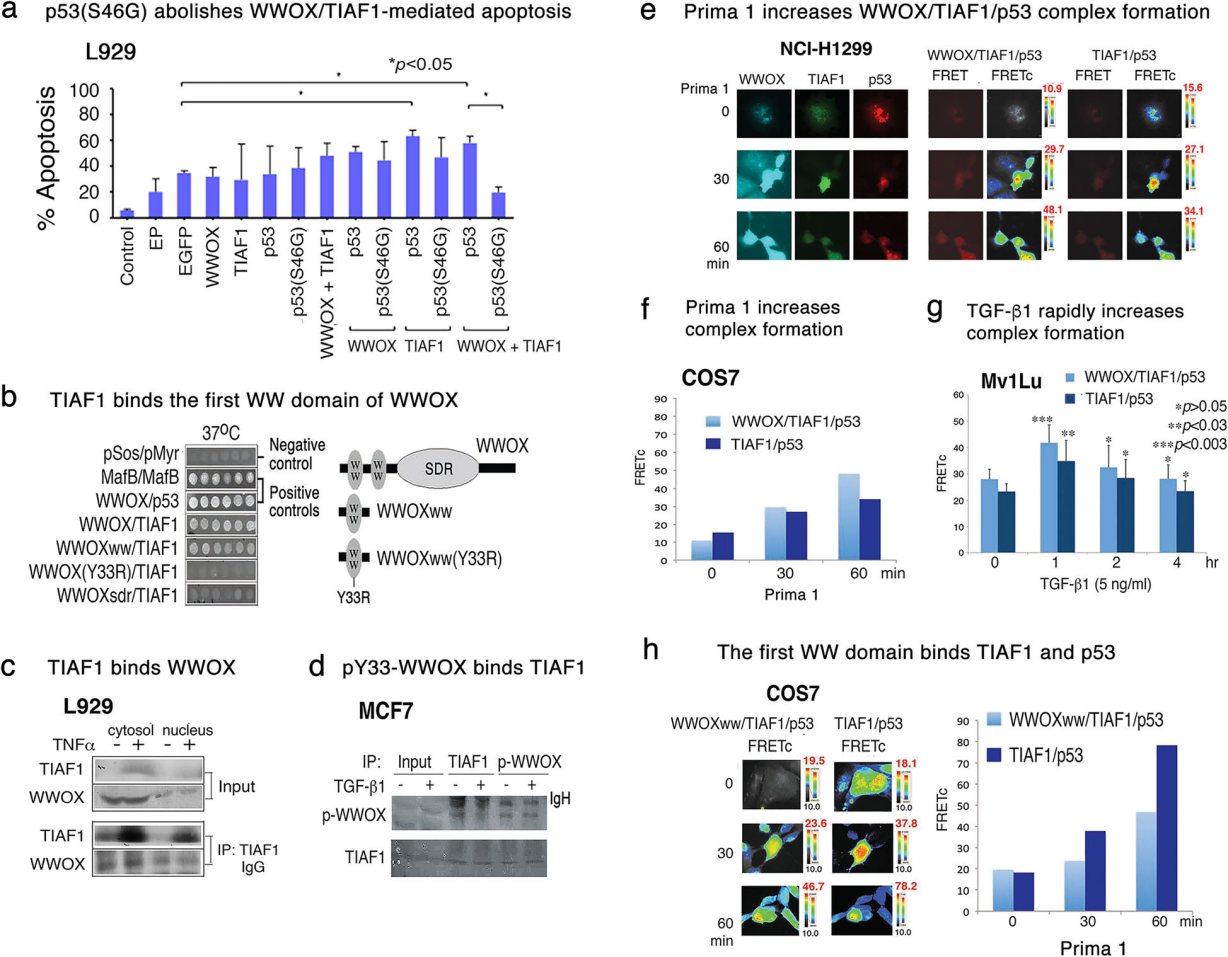
Transiently overexpressed WWOX/TIAF1/p53 triad induces apoptosis, and determination of the kinetics of the triad formation. **a** L929 cells were electroporated with indicated plasmids. Overexpression of WWOX, TIAF1 and p53 induced apoptosis. Dominant-negative p53 (S46G) suppressed apoptosis mediated by p53 and TIAF1. EP: electroporation with medium only (Mean ± S.D., *n* = 3, *p < 0.05, student’s *t* test). **b** Ras rescue-based yeast two-hybrid analysis revealed the binding of TIAF1 to the *N*-terminal first WW domain of WWOX, as shown by the growth of yeast at 37 °C [29–31]. WWOX(Y33R) mutant abolished the binding. The SDR domain failed to bind TIAF1. In positive binding controls, MafB self-association and WWOX/p53 binding are shown, and empty pSos versus empty pMyr as negative controls. cDNAs constructed in the pSos are shown in the left, and pMyr in the right. **c** TNFα, at 100 ng/ml, rapidly upregulated the expression of TIAF1 in 30 min in L929 cells. Co-immunoprecipitation showed the binding of TIAF1 with WWOX. **d** By co-immunoprecipitation, TGF-β1 (5 ng/ml) had little or no effect on the binding of TIAF1 with pY-WWOX during treatment for 30 min in MCF7 cells. IgH: IgG heavy chain. **e**, **f** NCI-H1299 cells were transiently transfected with p53-DsRed, TIAF1-EGFP, and WWOX-ECFP. By 3-protein FRET analysis [37, 39], Prima-1 induced p53 activation-dependent binding with WWOX and TIAF1 with time (see the number in red under FRETc columns, **e** Experiments were designed to let the FRET energy transfer from WWOX-ECFP to TIAF1-EGFP, and finally to p53-DsRed [37, 39]. Prima-1 increased the triad complex formation in COS7 cells (**f**). **g** In Mv1Lu cells, TGF-β1 rapidly increased the triad complex formation in 1 h, followed by reduction (*n* = 6; Student’s t test; experiments versus time 0 controls). **h** Prima-1 increased the binding of the first WW domain of WWOX with TIAF1 and p53 with time. Each bar represents an average of two experiments in (**f**) and (**h**)

Next, we investigated whether the overexpressed WWOX/TIAF1/p53 triad limits cell migration is due, in part, to induced apoptosis. In agreement with our previous observations [23], ectopic expression of the triad proteins induced apoptosis of L929 cells (Fig. 5a). Similar results were observed using other types of cells such as breast MDA-MB-231, COS7 fibroblasts, and lung NCI-H1299 cells. We have shown that pY33-WWOX physically binds pS46-p53, and the complex induces apoptosis [31]. More than 50% of cancer cells have p53 mutations and Ser46 is one of them [31, 43]. p53(S46G), which has an alteration of Ser46 to Gly, blocked the WWOX/TIAF1 complex-mediated apoptosis of L929 cells (see the last two columns to the right; Fig. 5a). However, p53(S46G) could not significantly suppress apoptosis induced by WWOX or TIAF1 alone (Fig. 5a). p53(S46G) does not bind WWOX [31].

### TIAF1 binds the first N-terminal WW domain of WWOX

We investigated how WWOX binds TIAF1. By Ras rescue-based Cytotrap yeast two-hybrid analysis [27, 29, 30, 34, 35, 38, 39], we determined the binding of TIAF1 to the *N*-terminal first WW domain of WWOX, as revealed by mutant yeast growth at 37 °C using a selective medium in agarose plates (Fig. 5b). When the conserved phosphorylation site Tyr33 is altered to Arg, WWOX(Y33R) could not bind TIAF1, suggesting that Tyr33 phosphorylation is involved. Also, the SDR domain of WWOX did not bind TIAF1. MafB self-association and WWOX/p53 binding showed the positive binding interactions (Fig. 5b). Empty vector pSos and empty vector pMyr did not yield in positive binding (Fig. 5b).

To further validate the TIAF1/WWOX binding, L929 cells were stimulated with TNFα for 30 min. TNFα rapidly increased TIAF1 protein expression (Fig. 5c). Binding of TIAF1 with WWOX was observed by co-immunoprecipitation. Also, endogenous TIAF1 binds Tyr33-phosphorylated WWOX (pY33-WWOX) in breast MCF7 cells, and that TGF-β1 had little or no effect on the binding during treatment for 30 min. (Fig. 5d). This data supports the observation from yeast two-hybrid analysis that pY33-WWOX binds TIAF1.

### Activated p53 binds TIAF1, and WWOX strengthens the p53/TIAF1 complex to stabilize the triad

Under physiologic conditions, TIAF1 does not bind p53 [40]. However, under stress conditions, both p53 and WWOX are activated. pS46-p53 physically binds pY33-WWOX and the complex translocates to the mitochondria or nucleus to induce apoptosis [29–31]. Here, p53-deficient NCI-H1299 cells were transiently transfected with p53-DsRed, TIAF1-EGFP, and WWOX-ECFP. Twenty-four hour later the cells were treated with Prima-1 for activating p53 [52]. By tri-molecular binding FRET analysis [37, 39], we measured the signal flow or the binding energy flow from WWOX-ECFP to TIAF1-EGFP and then to p53-DsRed [37]. Prima-1 increased the binding of p53 with TIAF1 and WWOX in a time-related manner (Fig. 5e; see the number in red under FRETc columns). Interestingly, the binding strength of the whole triad was stronger than that of the activated p53/TIAF1 complex. Similar results were observed by using p53-positive COS7 cells transfected with the aforementioned constructs and treated with Prima-1 (Fig. 5f). Similarly, TGF-β1 rapidly induced the triad complex formation in 1 h, followed by reduction in epithelial Mv1Lu cells (Fig. 5g). The Mv1Lu cell is sensitive to TGF-β-mediated growth suppression [53]. The binding strength of p53 and TIAF1 was relatively weak, compared to the overall protein binding strength in the triad.

Next, COS7 cells were transiently transfected with the first WW domain of WWOX (WWOXww), along with TIAF1 and p53. Again, Prima-1 induced the triad formation with time (Fig. 5h). Interestingly, the p53/TIAF1 complex had a greater binding strength than that of the overall binding strength in the triad complex. The observations suggest that activated p53 binds TIAF1, and WWOX joins the complex via its *C*-terminal SDR domain to stabilize the complex triad.

### p53 and TIAF1 block WWOX and Smad4-induced SMAD promoter activation

**Fig. 6:**
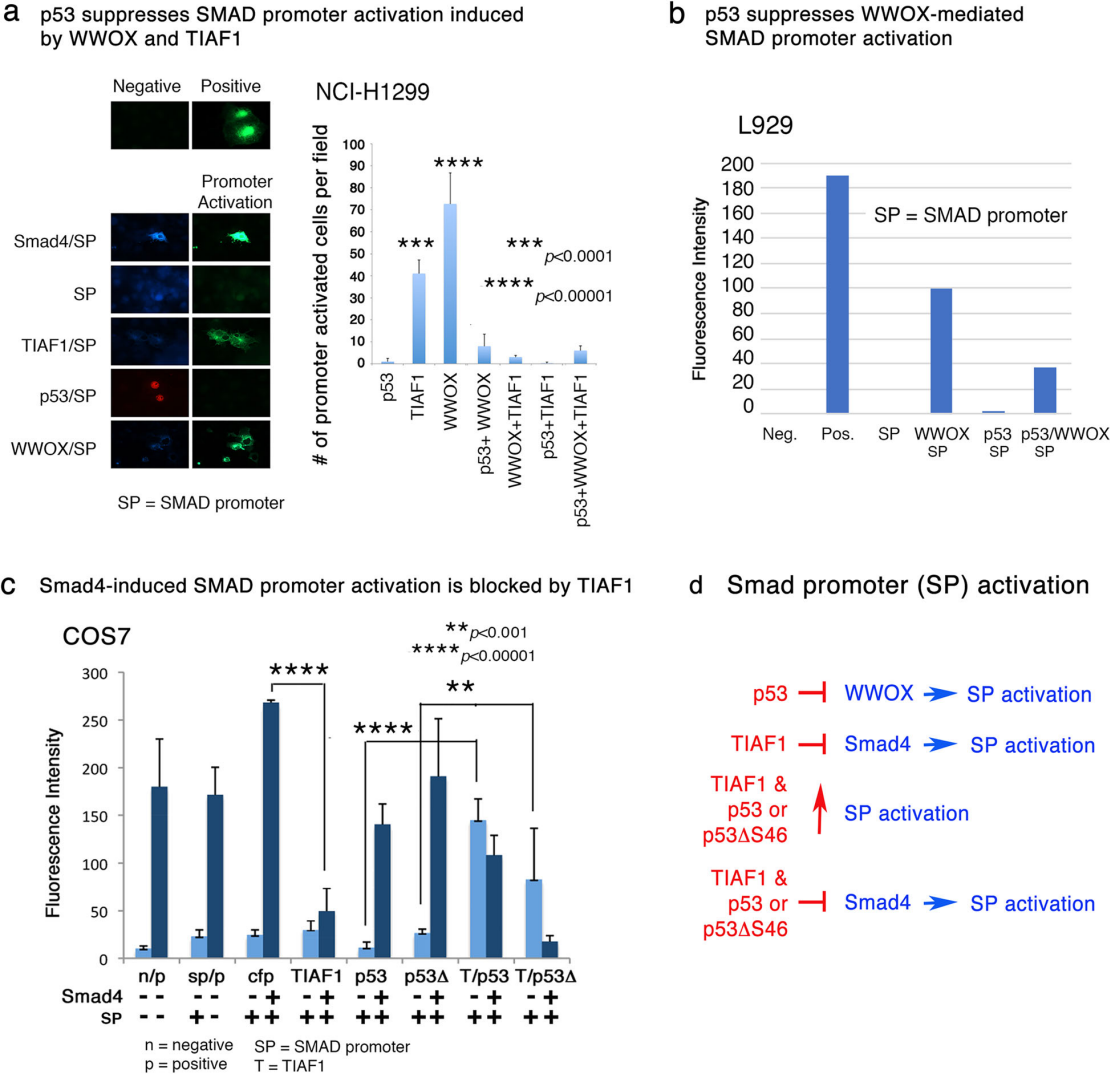
p53 abolishes WWOX-induced SMAD promoter activation. **a** NCI-H1299 cells were transiently overexpressed with TIAF1-ECFP, p53-DsRed, and/or WWOX-ECFP in the presence of a SMAD promoter plasmid with EGFP as a reporter. WWOX and TIAF1 induced the SMAD promoter activation, and that p53 abolished the activation. In a positive control, Smad4 induced the promoter activation. **b** In parallel, WWOX-induced Samd4 promoter activation was blocked by p53 in L929 cells. **c** Similarly, COS7 cells were transfected with Smad4-ECFP and the SMAD promoter construct, in the presence or absence of p53, p53ΔS46, and/or TIAF1. **d** In summary, data shows 1) p53 blocks WWOX-mediated SMAD promoter activation, 2) TIAF1 blocks Smad4-mediated SMAD promoter activation, 3) p53 or p53ΔS46 increases Smad4-mediated SMAD promoter activation, and 4) TIAF1 blocks p53 or p53ΔS46-increased Smad4-mediated SMAD promoter activation. SP = SMAD promoter

Suppressing one of the proteins in the triad abolished its tumor suppressor function [23] (Figs. 4 and 6), suggesting a concerted teamwork among p53, WWOX and TIAF1 is needed for cancer suppression. In light of the dynamic binding among the triad proteins (Fig. 5), we examined the potential functional antagonism among the triad proteins. p53-deficient lung cancer NCI-H1299 cells were transiently overexpressed with TIAF1-ECFP, p53-DsRed, and/or WWOX-ECFP, in the presence of the SMAD promoter construct. We determined that WWOX induced the SMAD promoter activation, and that p53 and/or TIAF1 abolished the activation (Fig. 6a). In a positive control, Smad4 induced the promoter activation (Fig. 6a; see left panel). Under similar conditions, p53 blocked WWOX-induced SMAD promoter activation in L929 cells (Fig. 6b). Furthermore, ectopic Smad4-mediated SMAD promoter activation was abolished by TIAF1 (Fig. 6c), which is in agreement with our previous report [23]. p53 and p53ΔS46 induced Smad4-dependent SMAD promoter activation (Fig. 6c). TIAF1 and p53 (or p53ΔS46) blocked Smad4-mediated SMAD promoter activation, and p53ΔS46 less effective (Fig. 6c). The entire molecular event is summarized (Fig. 6d). Together, our data showed that p53 and TIAF1 may functionally interrupt with WWOX in affecting SMAD-dependent promoter activation.

### WWOX suppresses cancer inflammation-mediated spleen enlargement and p53 abolishes the event, and the p53/WWOX antagonism-induced inflammation leading to brain protein aggregation

**Fig. 7:**
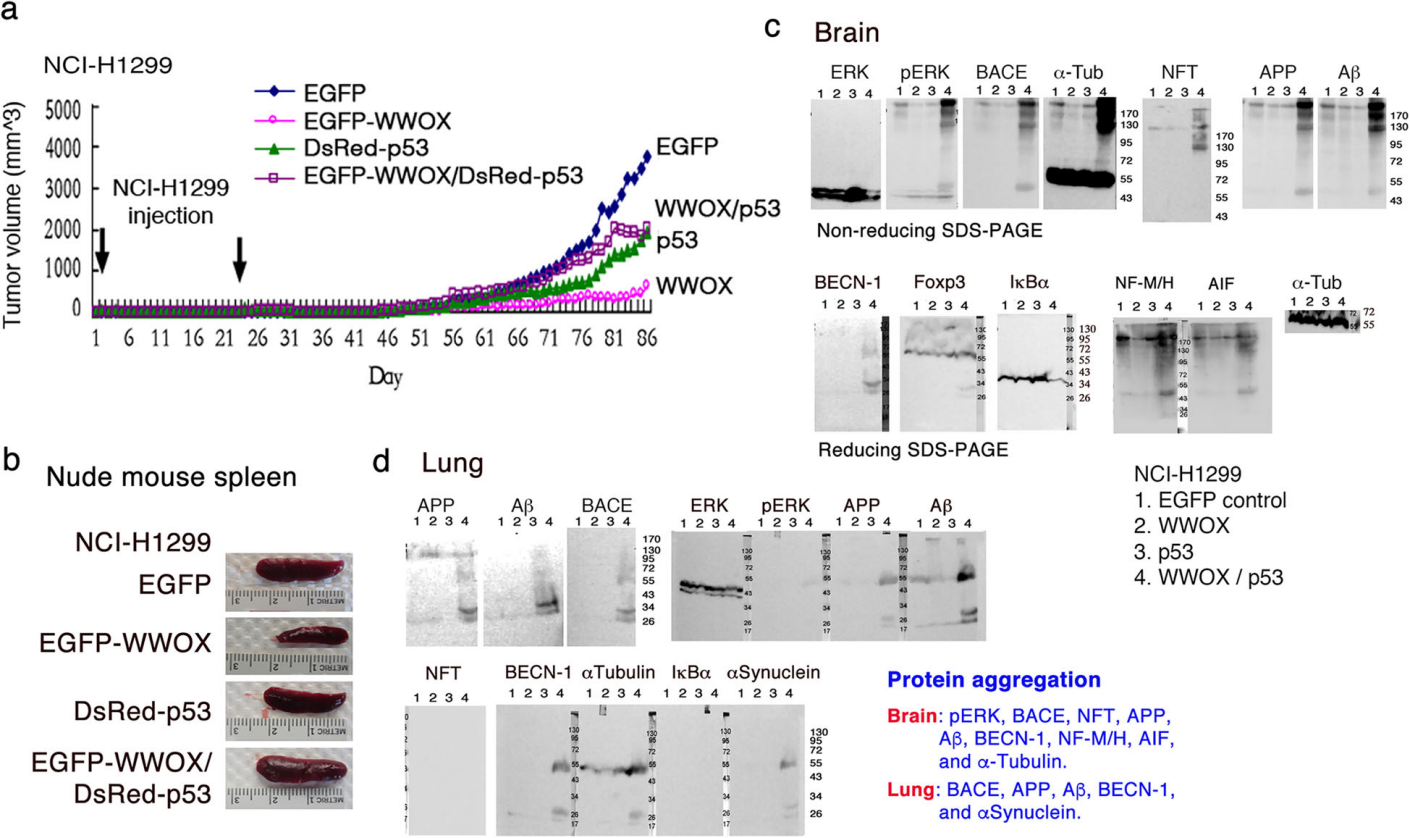
p53 counteracts WWOX-mediated suppression of NCI-H1299 cell growth in nude mice and this antagonism leads to neurodegeneraiton in vivo. **a** NCI-H1299 cells were transiently overexpressed with p53-DsRed and/or WWOX-ECFP, or EGFP only. Nude mice received subcutaneous injections of these cells twice on both sides of the flanks. Tumor sizes were measured daily. WWOX suppressed tumor growth, and the suppression was abolished by p53. A representative data is shown from two experiments. **b** Mice were sacrificed on day 86. Spleen enlargement occurred in all mice except in the mouse harboring WWOX-expressing NCI-H1299. **c** Protein aggregates were found for pERK, BACE, APP, Aβ, NFT and α-tubulin in the brain of mouse inoculated with p53/WWOX-expressing NCI-H1299 (top panel; nonreducing gels). APP was degraded. Under reducing conditions (bottom panel), aggregation of BECN-1, neurofilament NF-M/H, and AIF is shown. **d** Similar results were observed for the expression of BACE, APP, Aβ, BECN-1, and αSynuclein in the mouse lung

NCI-H1299 cells were transiently overexpressed with DsRed-p53 and/or EGFP-WWOX, or EGFP only (Fig. 7a), followed by inoculating the cells in subcutaneous sites of both flanks in nude mice. WWOX-NCI-H1299 cells had a suppressed growth in vivo. However, p53 abolished the growth suppression (Fig. 7a). Mice were sacrificed on day 86. Spleen enlargement was shown in mice inoculated with cells-expressing EGFP, DsRed-p53, or EGFP-WWOX/DsRed-p53 (Fig. 7b), suggesting that cancer-induced inflammation occurred. In contrast, WWOX blocked cancer cell-induced inflammation, so that the spleen size was the smallest (Fig. 7b). Notably, protein aggregates, including pERK, BACE, APP, Aβ, and NFT, were found in the brain of mice inoculated with p53/WWOX-expressing cells (Fig. 7c). Gels were run under both reducing and non-reducing conditions. These proteins are associated with neurodegeneration such as Alzheimer’s disease [22, 24–26]. Housekeeping protein α-tubulin was also aggregated. Additional proteins became aggregated were AIF and neurofilament middle and high molecular weights (NF-M/H) (Fig. 7c). We have recently reported the occurrence of neurodegeneration during the progression of melanoma in mice [26]. No protein aggregates were found in the mice inoculated with cells expressing EGFP, DsRed-p53, or EGFP-WWOX alone (Fig. 7c). APP was shown to be degraded. Similarly, upregulated BACE, APP degradation, and Aβ formation were also observed in the mouse lung (Fig. 7d). Aggregated BACE, APP, Aβ, BECN-1 and αSynuclein were shown in the lung.

## Discussion

In agreement with our recent report [54], WWOX-deficient cancer cells and *Wwox* gene knockout MEF cells have loose intercellular contacts and they migrate individually. Also, their migration speed is much faster than that of the wild type cells. This accounts for the aggressive behavior of metastatic cancer cells [14]. The JAK2/STAT3 axis has been implicated in the enhanced breast cancer cell metastasis [14]. In parallel, a recent study showed that WWOX restricts the migration of triple negative breast cancer cells via regulating the expression of miR-146a [55]. We showed that aberrant signal pathways occur in the *Wwox* knockout MEF cells. These cells possess constitutive activation of Smad3 and p38, along with significant downregulation of p53, IκBα, Smad6 and Fas. Whether these events contribute to enhanced cell proliferation of *Wwox* knockout MEF cells remains to be established. Indeed, the knockout cells tend to undergo apoptosis post rapid proliferation (data not shown).

The wild type cells have strong cell-cell contacts and migrate collectively. WWOX undergoes Tyr33 phosphorylation and nuclear translocation upon stimulating cells with TGF-β [34]. TGF-β accelerates the collective migration of wild type MEF cells. Also, there is an increased cell number migrating individually, supporting the role of TGF-β in promoting cancer cell migration and metastasis [34, 48]. By co-culturing the wild type and *Wwox* knockout MEF cells, the knockout cells always undergo retrograde migration upon facing the wild type cells [54]. Multiple signal pathways, including MIF, Hyal-2, Eph, and Wnt pathways that converge to ERK signaling is responsible for the retrograde migration [54].

We continued to show that the p53/TIAF1/WWOX axis is a protein triad for cancer suppression, including inhibition of cell migration, adherence-independent growth, and SMAD promoter activation, and induction of cancer cell apoptosis [23]. p53, TIAF1 and WWOX act in a concerted manner to inhibit cancer cell growth, migration and apoptosis in vitro. Missing a component in the p53/TIAF1/WWOX triad reduces its cancer inhibitory function. WWOX, via its first *N*-terminal WW domain, physically binds p53 and TIAF1, suggesting that p53 and TIAF1 competitively bind WWOX. Under apoptotic stress, pY33-WWOX binds pS46-p53 to carry out apoptosis [31]. Binding of TIAF1 with WWOX depends upon phosphorylation of WWOX at Tyr33. TIAF1 undergoes phosphorylation Ser37 [22, 23, 28]. Whether pS37-TIAF1 binds pY33-WWOX is unknown. Like p53 and WWOX, TIAF1 is significantly downregulated or could be altered in many types of cancer cells [23, 28, 56].

Under physiologic conditions, the binding strength for p53, WWOX and TIAF1 is weak. p53 fails to bind TIAF1 [53]. Intriguingly, Prima-1 activates p53, and this leads to the p53/TIAF1 complex formation. WWOX appears to further enhances the binding with p53/TIAF1 to increase the stability of the triad. The SDR domain of WWOX is probably involved in the triad stabilization. Whether TIAF1 is Ser37 phosphorylated in the triad remains to be determined. Both TNFα and TGF-β1 rapidly increase the triad formation, further supporting the observations that the p53/TIAF1/WWOX triad participates in cancer growth suppression, migration inhibition, and apoptosis. Quite frequently, when two proteins bind reaching a maximal strength, the protein complex further drives to polymerization and leads to aggregation for causing apoptosis [22–24, 28]. Thus, rapid triad formation of p53, TIAF1 and WWOX is important for cancer suppression and causing cancer cell death. Δ133p53γ isoform strongly suppresses cancer cell migration, and this positively correlates with its-mediated SMAD promoter activation. We do not exclude the possibility that Δ133p53γ undergoes self-association and this provides a driving energy to cause SMAD promoter activation and inhibition of cell migration. The molecular nature of the Δ133p53γ isoform in cancer cells remains to be established.

p53 and WWOX are known to induce apoptosis in a synergistic manner [29–31]. We demonstrated for the first time that there is a functional antagonism between these two tumor suppressors. For example, p53 blocks WWOX-induced SMAD promoter activation. Notably, WWOX-overexpressing NCI-H1299 cells do not elicit inflammatory immune response. However, p53 abolishes the function of WWOX in causing cancer-mediated inflammation. This functional antagonism correlates with the increased tumor growth and neurodegeneration in vivo, as shown by growing p53/WWOX-expressing NCI-H1299 cells in nude mice. WWOX inhibition of NCI-H1299 tumor growth is blocked by p53. For unknown reasons, the growing tumors induce protein aggregation in the mouse brain and lung. We believe that cytokines released by growing cancer cells induce protein aggregation. These aggregated proteins include pERK, BACE, α-tubulin, NFT, APP and Aβ, in which BACE upregulation, APP degradation, and Aβ formation occur in the brain. BACE is responsible for cleaving APP that allows formation of Aβ formation and plaque generation. The observations indicate there is an ongoing neurodegeneration in the brain of tumor-growing mice. We have recently shown that growing melanoma or glioblastoma in mice leads to neurodegeneration [26]. Downregulation of WWOX in the brain may lead to a cascade of protein aggregation, starting from TRAPPC6AΔ, TIAF1, and SH3GLB2, which results in APP degradation, and aggregation of amyloid β and tau [3, 23–26]. How the tumor cells in the flanks control neurodegeneration in the brain is unknown and remains to be established.

TIAF1 is a potential tumor suppressor. We demonstrated the anticancer function of TIAF1 by showing its critical role in cell death. TIAF1 rapid interaction with WWOX is essential in executing cell death. TIAF1 is upregulated in growing tumors, but may disappear in established metastatic cancer cells [23, 28]. TIAF1 protein aggregation has been shown in the human cortex and hippocampus of nondemented mid-aged humans and demented old patients [22, 24–26]. TIAF1 aggregates, together with Smad4 and Aβ, are found in the cancer stroma and peritumor capsules of many solid tumors [23]. Presence of TIAF1/Aβ aggregates is shown on the interface between brain neural cells and the metastatic cancer cell mass. The TIAF1/Aβ aggregates is toxic to neural cells but not cancer cells. Also, TIAF1 and amyloid fibrils are significantly accumulated in the stroma of progressing lung cancer cells [23]. These peritumor materials probably provide support for cancer cell survival.

TIAF1 undergoes self-association, which leads to increased expression of Smad4 and WWOX [23]. WWOX in turn increases the TIAF1 expression. Binding of TIAF1 with Smad4 induces Aβ formation [23]. TIAF1 suppresses SMAD-regulated promoter activation. Notably, in the absence of p53, self-aggregating TIAF1 spontaneously activated the SMAD-regulated promoter. pS15-p53 requires the presence of TIAF1 to undergo nuclear translocation [40]. Together, our previous and current findings imply that Smad4 and p53 restrict TIAF1 self-aggregation, and that loss of tumor suppressors p53, WWOX, and Smad4 results in TIAF1 aggregate formation, which supports cancer growth and causes neurodegeneration.

## Conclusions

**Fig. 8:**
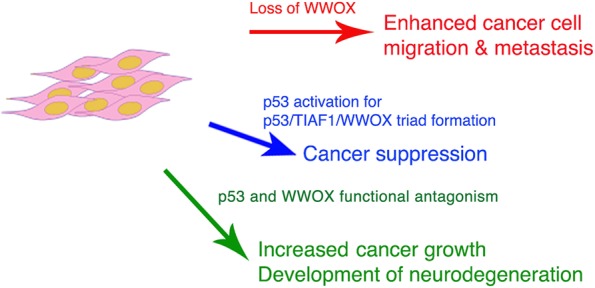
Summary illustration. Three scenarios account for cancer growth or suppression: 1) loss of WWOX, p53 and TIAF1 increases cancer growth and metastasis; 2) stabilized p53/WWOX/TIAF1 triad suppresses cancer growth, inhibits metastasis, and induces apoptosis; 3) functional antagonism between p53 and WWOX allows cancer cell growth and yet induces inflammation for causing neural protein aggregation as seen in the Alzheimer’s disease

In conclusion, we have provided strong evidence for the binding interactions among WWOX, TIAF1 and p53, and the firmly established WWOX/TIAF1/p53 protein triad exerts strong cancer suppression by blocking cancer cell migration, anchorage-independent growth and SMAD promoter activation, and inducing apoptosis (see Summary illustration, Fig. 8). Yet, p53 may functionally antagonize with WWOX. p53 blocks WWOX inhibition of inflammatory immune response induced by cancer, and this leads to protein aggregation in the brain as seen in the Alzheimer’s disease and other neurodegeneration. pS14-WWOX is known to be associated with the severe progression of cancer and neurodegeneration [1, 26]. Upregulation of pS14-WWOX in the lesions of cancer and neurodegeneration correlates with downregulation of pY33-WWOX. Apparently, pS14-WWOX favors inflammatory response for disease progression. Whether p53 binds pS14-WWOX to promote diseases is unknown.

## Additional files

Additional file 1: **Figure S1.** Wild type MEF cells migrate collectively, whereas Wwox knockout MEF cells migrate individually. Shown is the imaging of cell migration at 0 and 48 h by time-lapse microscopy. Also, see Additional file 2: Video S1 and Additional file 3: Video S2. The image is digitally enlarged from Fig. 2d. **Figure S2.** WWOX-deficient cells have a faster migration rate. WWOX-deficient MDA-MB43 5 s and MDA-MB-231 migrated faster than WWOX-positive L929 s cells. WWOX appears to be functionally deficient in MCF7 cells, as these cells migrated effectively compared to the WWOXnegative cells. This data links to Fig. 2. **Figure S3.** TGF-β1 suppresses the migration of Wwox knockout cells. MEF cells were treated with TGF-β1 or TGF-β2 (10 ng/ml) for 48 h. Both TGF-β1 and TGF-β2 promoted wild type cell migration. TGF-β2 is more effective in suppressing the knockout cell migration than TGF-β1. This data links to Fig. 2i. **Figure S4.** TGF-β1 does not affect the proliferation of Wwox MEF cells. TGF-β1 had no significant effect on the cell proliferation of both wild type and the Wwox knockout MEF cells (*n* = 3, Student’s t test). This data links to Fig. 2. **Figure S5.** Colocalization of transiently overexpressed TIAF1 with p53 and WWOX proteins. MDA-MB-231 cells were transiently overexpressed with p53-DsRed, TIAF1-EGFP and WWOX-ECFP. TIAF1 underwent polymerization and retained p53 and WWOX in the cytoplasm (see punctate). The image data is enlarged from Fig. 3c. **Figure S6.** p53 is more potent than WWOX in blocking anchorage-independent cell growth. L929 cells were transfected with the following cDNA expression constructs for the anchorage-independent growth assay: 1) p53, 2) WWOX (OXFL), 3) p53ΔS46 (p53Δ46), 4) p53/WWOX, and 5) p53ΔS46/WWOX. Data is shown as an average of duplicate experiments. This data supports Fig. 3d and e. (PDF 8481 kb)

Additional file 2: **Video S1.** Migration of wild type *Wwox*^*+/+*^ MEF cells as determined by time-lapse microscopy. Wild type *Wwox*^*+/+*^ MEF cells were cultured in the right and left chambers of a culture-insert (ibidi) for 24 h. Following removal of the culture-insert, cells were allowed to migrate to each other from both sides. Time-lapse microscopy was performed at 37 °C with 5% CO2. (MP4 2361 kb)

Additional file 3: **Video S2.** Migration of knockout *Wwox*^*−/−*^ MEF cells as determined by time-lapse microscopy. Knockout *Wwox* ^*−/−*^MEF cells were cultured in the right and left chambers of a culture-insert (ibidi) for 24 h. Following removal of the culture-insert, cells were allowed to migrate to each other from both sides. Time-lapse microscopy was performed at 37 °C with 5% CO2. (MP4 3411 kb)

## Data Availability

All data generated or analyzed during this study are included in this article and the supplementary material.

## Abbreviations

ADH/SDR: Short chain alcohol dehydrogenase/reductase
AIF: Apoptosis-inducing factor
APP: Amyloid Precursor Protein
Aβ: Amyloid beta
BACE: Beta-secretase 1
BECN-1: Beclin-1
DMEM: Dulbecco’s modified eagle medium
FBS: Fetal bovine serum
*Foxp3*: Forkhead box P3
Hyal-2: Hyaluronidase type 2
IκBα: Inhibitor of nuclear factor kappaB alpha
MEF: Mouse embryonic fibroblast
NF-M/H: Neurofilament middle and high molecular weights (M and H)
NFT: *Neurofibrillary tangles*
NLS: Nuclear localization signal
NOS2: Nitric oxide synthase 2
NSYK motif: Asparagine-serine-tyrosine-lysine motif
PBS: Phosphate buffer saline
pERK: Phosphorylated extracellular signal–regulated kinases
SCC: Skin squamous cell carcinoma
SH3GLB2: SH3 Domain Containing GRB2 Like, Endophilin B2
TGF-β1: Transforming growth factor-β1
TIAF1: TGF-β1-induced antiapoptotic factor
TRAPPC6AΔ: Trafficking protein particle complex 6A delta
WWOX or WOX1: WW domain-containing oxidoreductase
