# Supplementary material for: A p53/TIAF1/WWOX triad exerts cancer suppression but may cause brain protein aggregation due to p53/WWOX functional antagonism
Source: Cell Commun Signal. 2019 Jul 17;17:76. doi: 10.1186/s12964-019-0382-y (PMC6637503; doi:10.1186/s12964-019-0382-y)

Figure S1. Wild type MEF cells migrate collectively, whereas *Wwox* knockout MEF cells migrate individually. Shown is the imaging of cell migration at 0 and 48 hr by time-lapse microscopy. Also, see Videos S1 and S2. The image is digitally enlarged from Figure 2d.

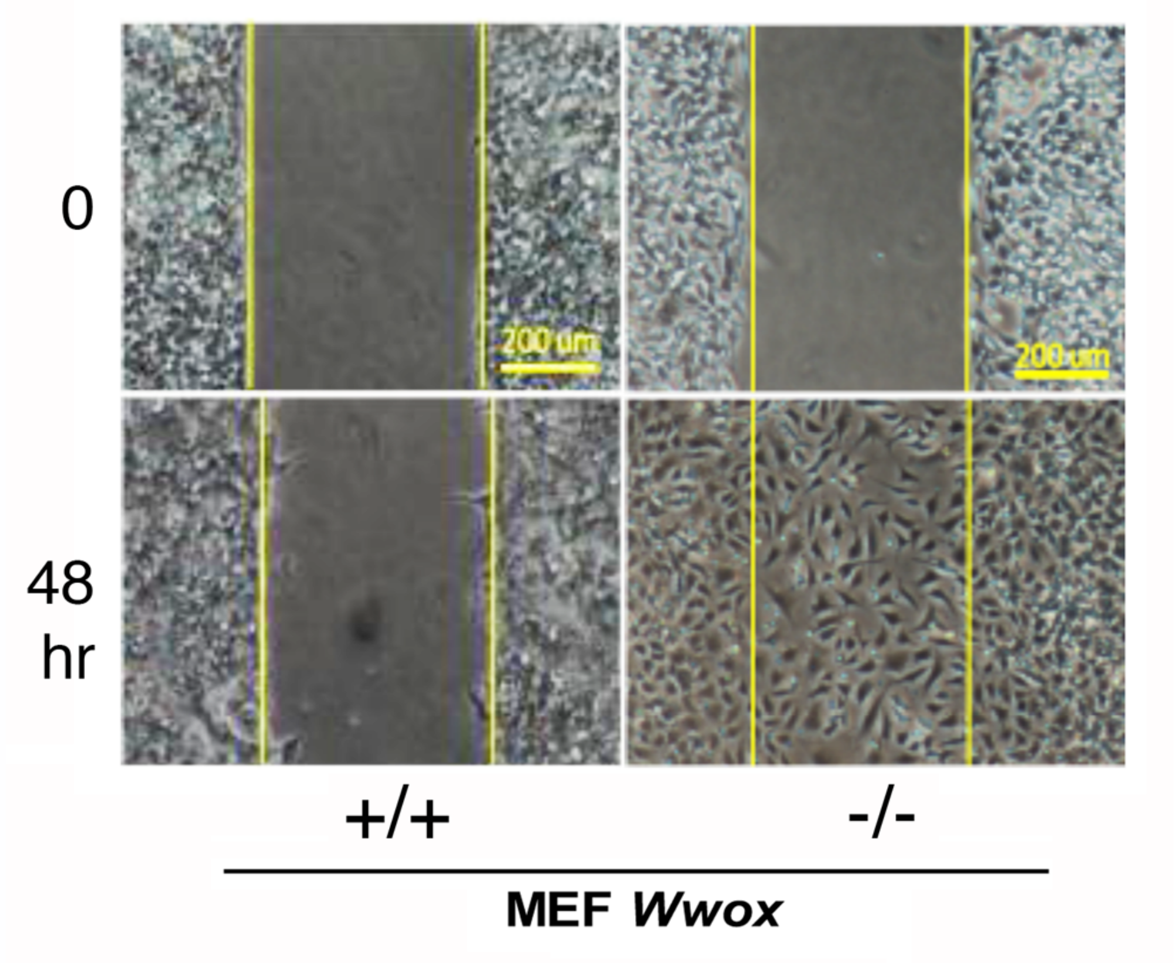

Figure S2. WWOX-deficient cells have a faster migration rate. WWOX-deficient MDA-MB-435s and MDA-MB-231 migrated faster than WWOX-positive L929s cells. WWOX appears to be functionally deficient in MCF7 cells, as these cells migrated effectively compared to the WWOX-negative cells. This data links to Figure 2.

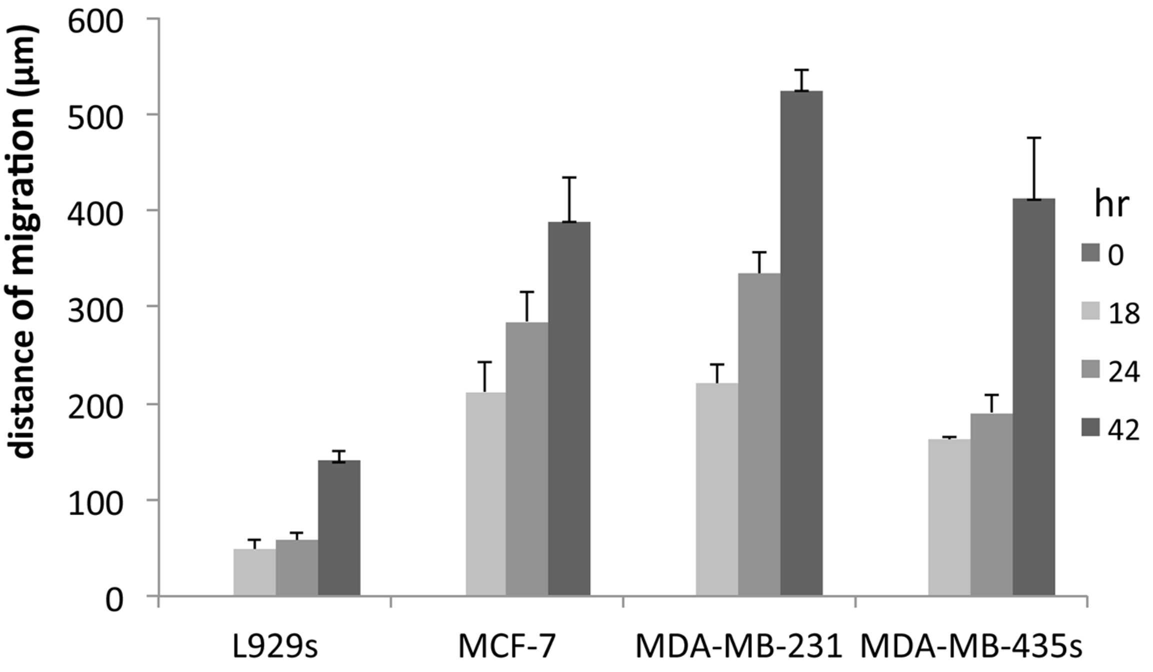

Figure S3. TGF-β1 suppresses the migration of *Wwox* knockout cells. MEF cells were treated with TGF-β1 or TGF-β2 (10 ng/ml) for 48 hours. Both TGF-β1 and TGF-β2 promoted wild type cell migration. TGF-β2 is more effective in suppressing the knockout cell migration than TGF-β1. This data links to Figure 2i.

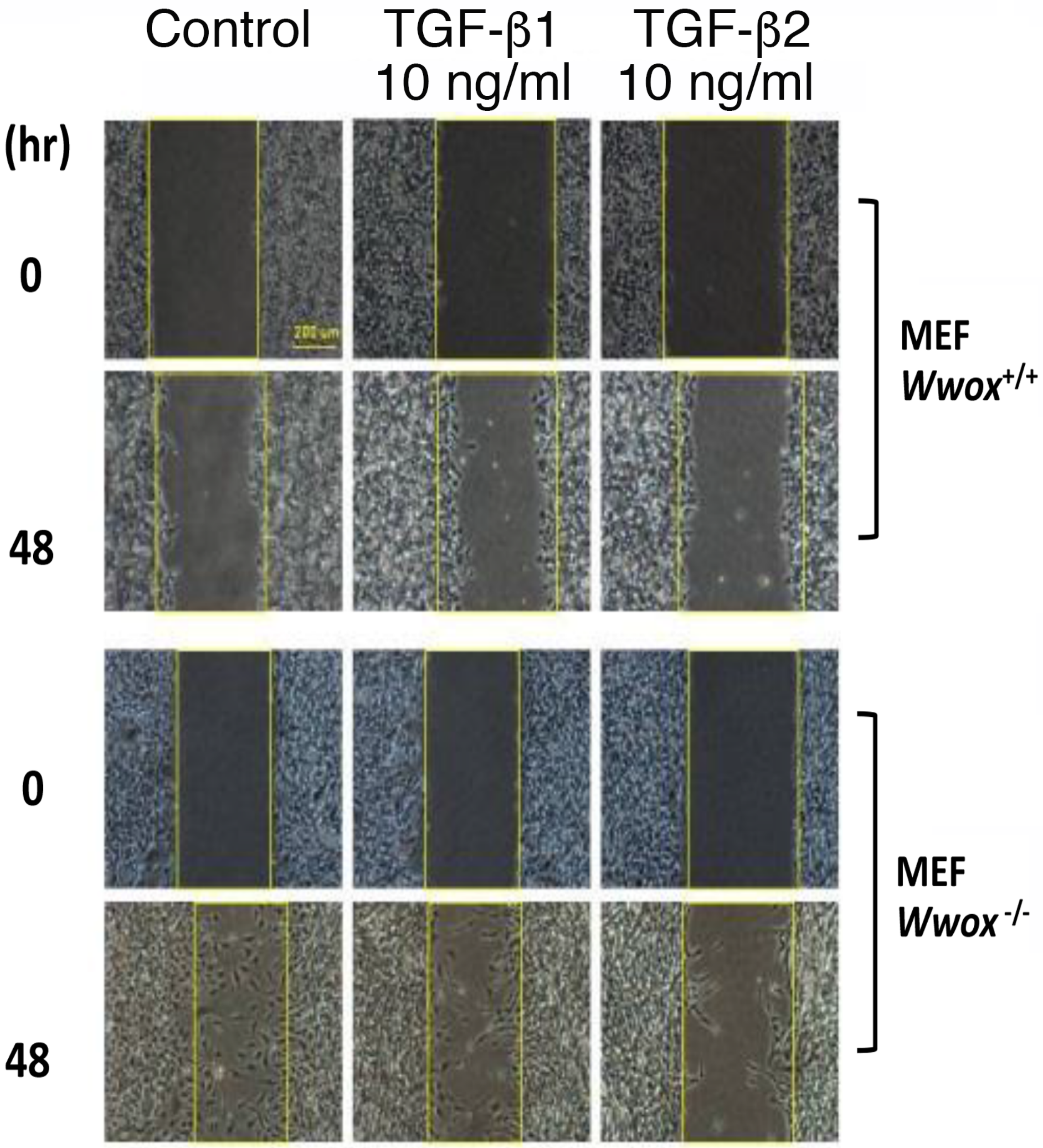

Figure S4. TGF-β1 does not affect the proliferation of *Wwox* MEF cells. TFG-β1 had no significant effect on the cell proliferation of both wild type and the *Wwox* knockout MEF cells (n=3, Student's *t* test). This data links to Figure 2.

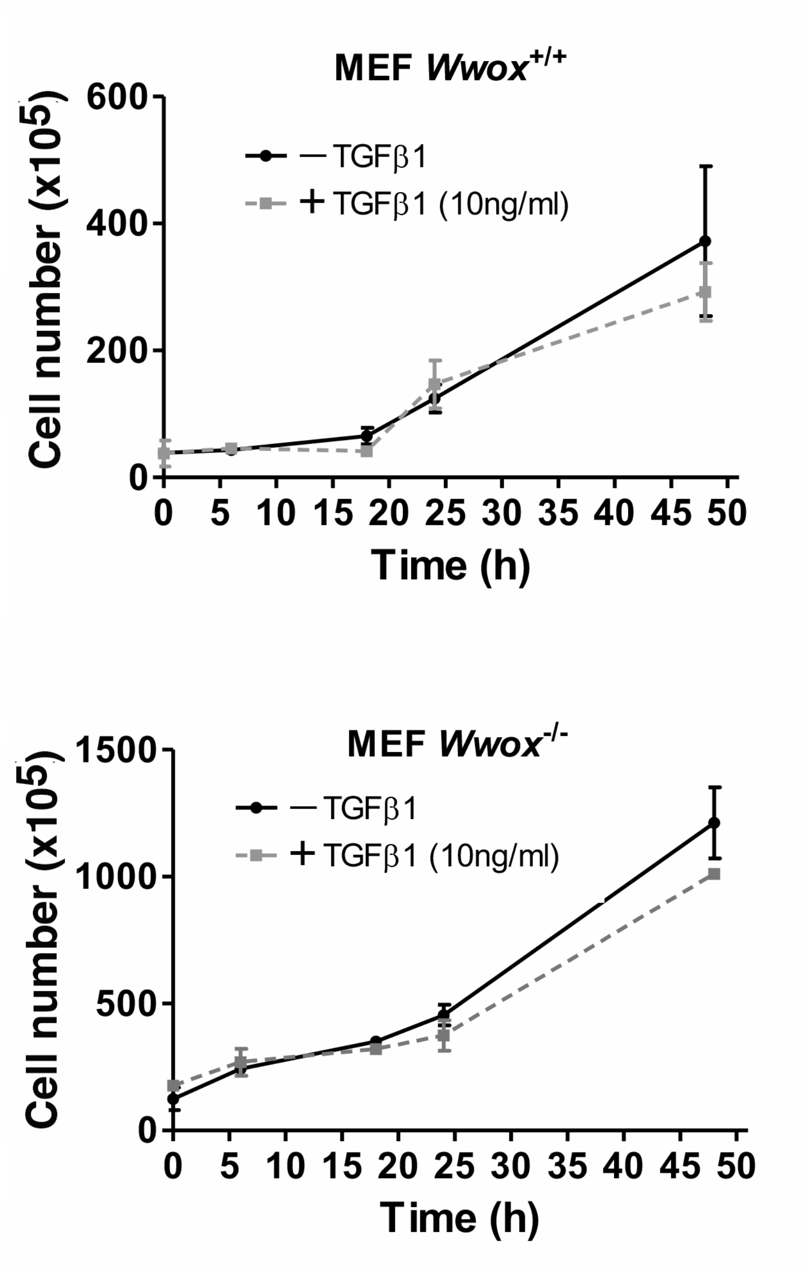

Figure S5. Colocalization of transiently overexpressed TIAF1 with p53 and WWOX proteins. MDA-MB-231 cells were transiently overexpressed with p53-DsRed, TIAF1-EGFP and WWOX-ECFP. TIAF1 underwent polymerization and retained p53 and WWOX in the cytoplasm (see punctate). The image data is enlarged from Figure 3c.

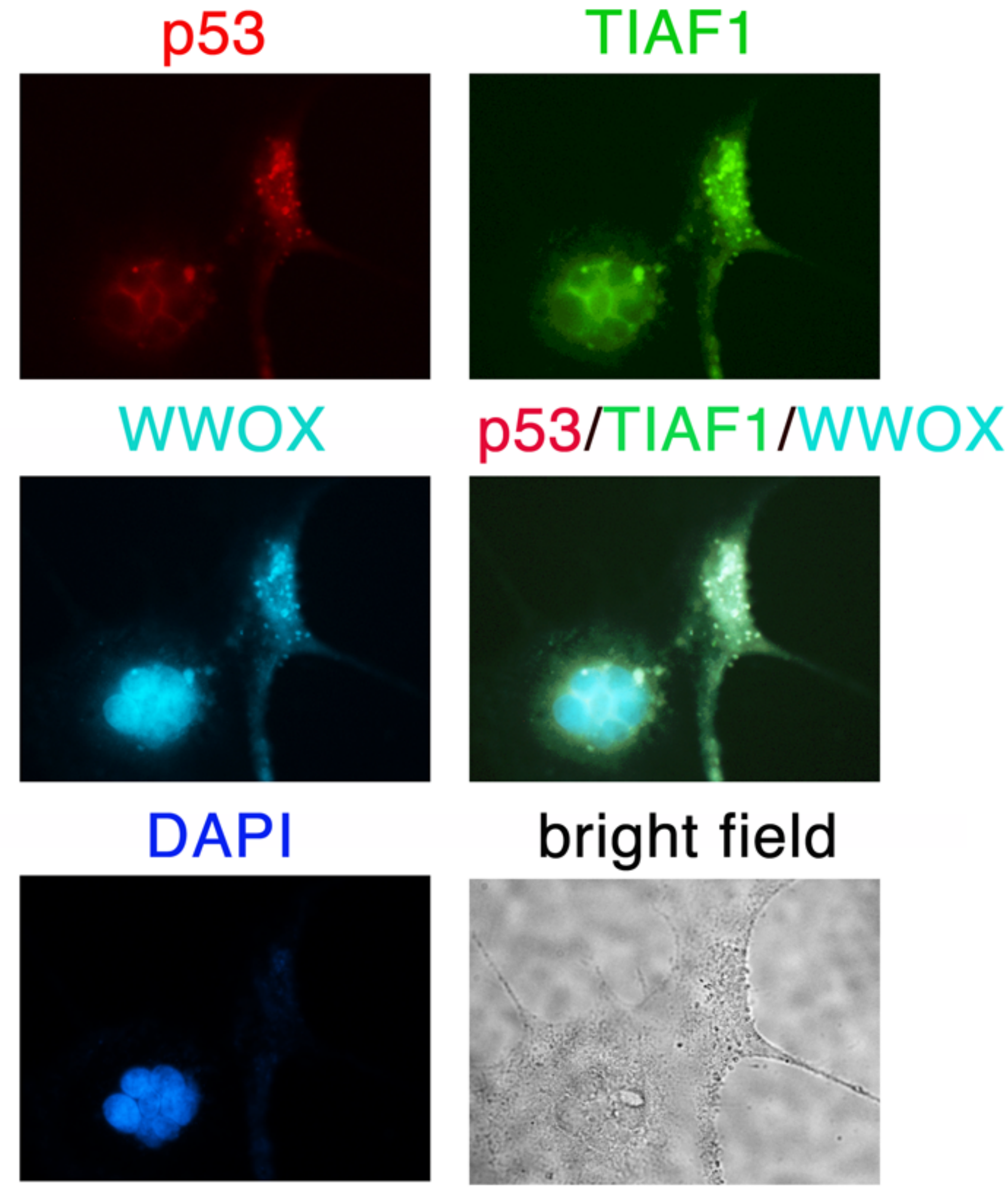

Figure S6. p53 is more potent than WWOX in blocking anchorage-independent cell growth. L929 cells were transfected with the following cDNA expression constructs for the anchorage-independent growth assay: 1) p53, 2) WWOX (OXFL), 3) p53ΔS46 (p53Δ46), 4) p53/WWOX, and 5) p53ΔS46/WWOX. Data is shown as an average of duplicate experiments. This data supports Figure 3d and e.

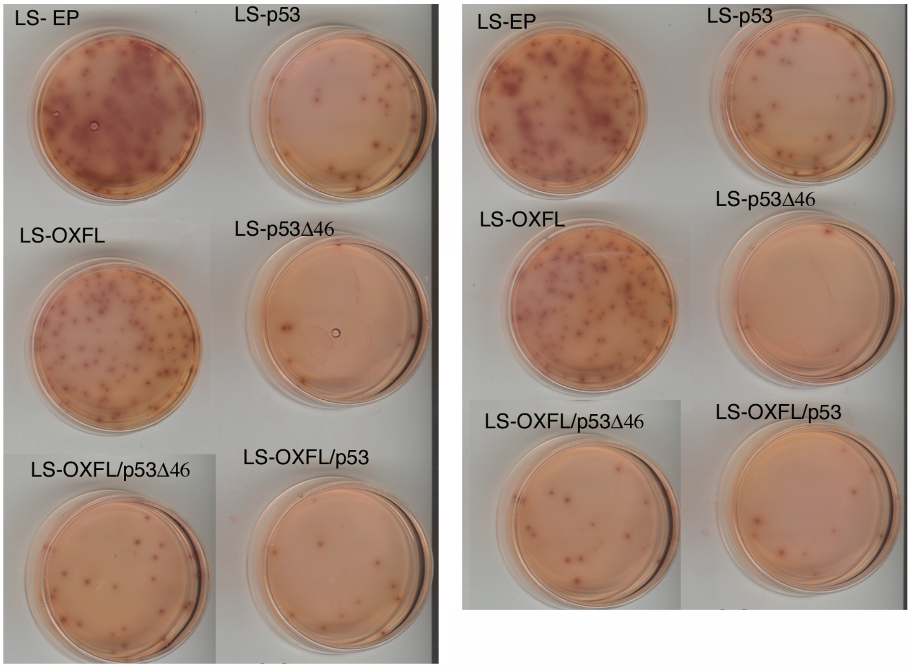

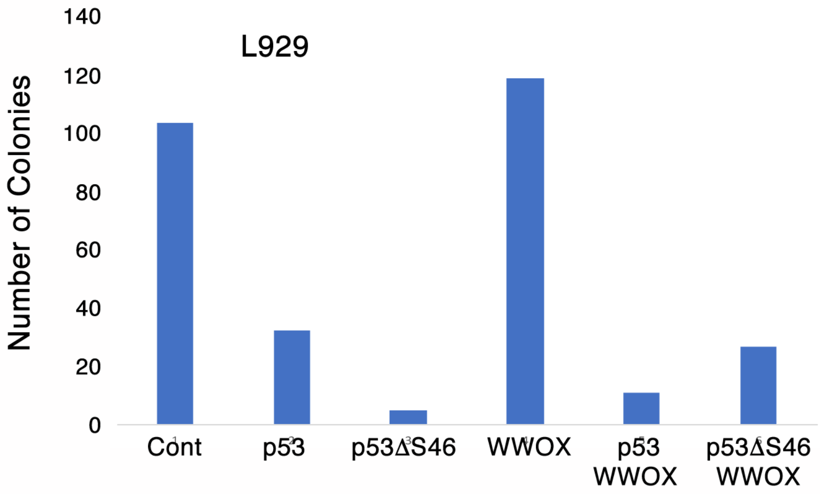

Supplement: Supplementary file 1 — Figure S1. Wild type MEF cells migrate collectively, whereas Wwox knockout MEF cells migrate individually. Shown is the imaging of cell migration at 0 and 48 h by time-lapse microscopy. Also, see Additional file 2: Video S1 and Additional file 3: Video S2. The image is digitally enlarged from Fig. 2d. Figure S2. WWOX-deficient cells have a faster migration rate. WWOX-deficient MDA-MB43 5 s and MDA-MB-231 migrated faster than WWOX-positive L929 s cells. WWOX appears to be functionally deficient in MCF7 cells, as these cells migrated effectively compared to the WWOXnegative cells. This data links to Fig. 2. Figure S3. TGF-β1 suppresses the migration of Wwox knockout cells. MEF cells were treated with TGF-β1 or TGF-β2 (10 ng/ml) for 48 h. Both TGF-β1 and TGF-β2 promoted wild type cell migration. TGF-β2 is more effective in suppressing the knockout cell migration than TGF-β1. This data links to Fig. 2i. Figure S4. TGF-β1 does not affect the proliferation of Wwox MEF cells. TGF-β1 had no significant effect on the cell proliferation of both wild type and the Wwox knockout MEF cells (n = 3, Student’s t test). This data links to Fig. 2. Figure S5. Colocalization of transiently overexpressed TIAF1 with p53 and WWOX proteins. MDA-MB-231 cells were transiently overexpressed with p53-DsRed, TIAF1-EGFP and WWOX-ECFP. TIAF1 underwent polymerization and retained p53 and WWOX in the cytoplasm (see punctate). The image data is enlarged from Fig. 3c. Figure S6. p53 is more potent than WWOX in blocking anchorage-independent cell growth. L929 cells were transfected with the following cDNA expression constructs for the anchorage-independent growth assay: 1) p53, 2) WWOX (OXFL), 3) p53ΔS46 (p53Δ46), 4) p53/WWOX, and 5) p53ΔS46/WWOX. Data is shown as an average of duplicate experiments. This data supports Fig. 3d and e. (PDF 8481 kb) [file 12964_2019_382_MOESM1_ESM.pdf]
